# Li_2_PtF_6_ revisited

**DOI:** 10.1107/S1600536814015566

**Published:** 2014-07-11

**Authors:** Florian Kraus

**Affiliations:** a AG Fluorchemie, Technische Universität München, Lichtenbergstrasse 4, 85747 Garching, Germany

**Keywords:** Lithium, platinum, fluoride, trirutile-type, crystal structure

## Abstract

In comparison with previous stucture determinations of Li_2_PtF_6_, dilithium hexa­fluorido­platinate(IV) [Graudejus *et al.* (2000 ▶). *Inorg. Chem.* **39**, 2794–2800; Henkel & Hoppe (1968 ▶). *Z. Anorg. Allg. Chem.* **359**, 160–177], the current study revealed the Li atom to be refined with anisotropic displacement parameters, thus allowing for a higher overall precision of the model. Li_2_PtF_6_ adopts the trirutile structure type with site symmetries of 2.*mm*, *m.mm*, ..*m* and *m*.2*m* for the Li, Pt and the two F sites. The Pt—F distances in the slightly distorted PtF_6_ octa­hedron are essentially similar with 1.936 (4) and 1.942 (6) Å, and the equatorial F—Pt—F angles range from 82.2 (2) to 97.8 (2)°. The Li—F distances in the somewhat more distorted LiF_6_ octa­hedron are 1.997 (15) and 2.062 (15) Å, with equatorial F—Li—F angles ranging from 76.3 (7) to 99.71 (17)°.

## Related literature   

Henkel & Hoppe (1968 ▶) reported on the synthesis of Li_2_PtF_6_ by direct fluorination of (NH_4_)_2_PtCl_6_ and Li_2_CO_3_. The obtained yellow Li_2_PtF_6_ was characterized by powder X-ray diffraction and reported to crystallize in the monoclinic crystal system. Graudejus *et al.* (2000 ▶) obtained Li_2_PtF_6_ in the form of yellow and air-stable crystals from the reaction of LiF with Pt in anhydrous HF under UV-photolysis of F_2_. The reported space group and unit cell parameters are in accordance with the current redetermination. However, a low precision of the Pt—F bond lengths of only ±0.01 Å was obtained due to many unobserved reflections even at the 2σ level. For synthetic details for the preparation of PtF_4_, see: Müller & Serafin (1992 ▶).

## Experimental   

### 

#### Crystal data  

Li_2_PtF_6_
*M* *_r_* = 322.97
Tetragonal, 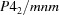

*a* = 4.6427 (1) Å
*c* = 9.1234 (2) Å
*V* = 196.65 (1) Å^3^
*Z* = 2
Mo *K*α radiation
μ = 35.71 mm^−1^
*T* = 150 K
0.05 × 0.05 × 0.04 mm

#### Data collection  

Oxford Diffraction Xcalibur3 diffractometer
Absorption correction: multi-scan (*CrysAlis RED*; Oxford Diffraction, 2007 ▶) *T*_min_ = 0.148, *T*_max_ = 1.000
5829 measured reflections
257 independent reflections
184 reflections with *I* > 2σ(*I*)
*R*_int_ = 0.062

#### Refinement  

*R*[*F* ^2^ > 2σ(*F* ^2^)] = 0.019
*wR*(*F* ^2^) = 0.052
*S* = 1.17
257 reflections
19 parameters
Δρ_max_ = 2.51 e Å^−3^
Δρ_min_ = −1.33 e Å^−3^

### 

Data collection: *CrysAlis CCD* (Oxford Diffraction, 2007 ▶); cell refinement: *CrysAlis RED* (Oxford Diffraction, 2007 ▶); data reduction: *CrysAlis RED*; program(s) used to solve structure: *SHELXS97* (Sheldrick, 2008 ▶); program(s) used to refine structure: *SHELXL97* (Sheldrick, 2008 ▶); molecular graphics: *DIAMOND* (Brandenburg, 2007 ▶); software used to prepare material for publication: *SHELXL97*.

## Supplementary Material

Crystal structure: contains datablock(s) I. DOI: 10.1107/S1600536814015566/wm5032sup1.cif

Structure factors: contains datablock(s) I. DOI: 10.1107/S1600536814015566/wm5032Isup2.hkl

CCDC reference: 1012012

Additional supporting information:  crystallographic information; 3D view; checkCIF report

## Figures and Tables

**Fig. 1.**
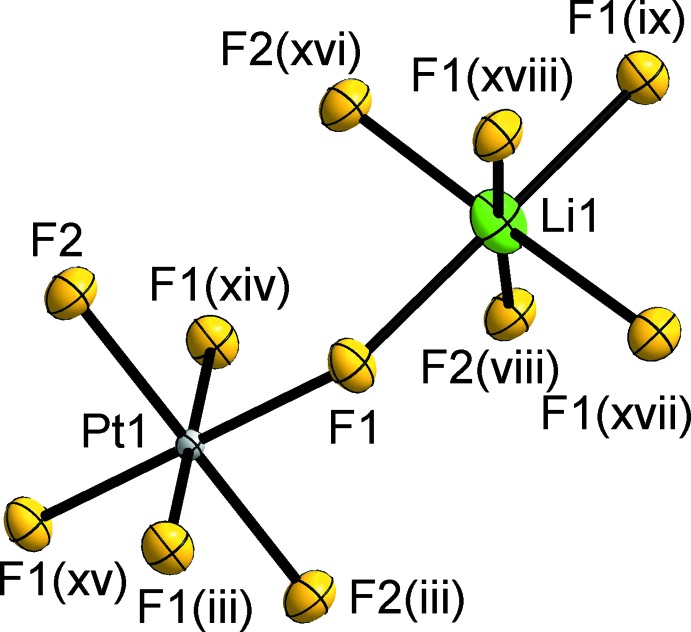
View of the coordination polyhedra around Pt and Li. Displacement ellipsoids are shown at the 70% probability level. [Symmetry codes: (iii) -x, -y, z; (viii) x + 1, y, z; (ix) -x + 1, -y + 1, z; (xiv) y, x, -z; (xv) -y, -x, -z; (xvi) -x, 1 - y, z; (xvii) x + 1/2, -y + 1/2, -z + 1/2; (xviii) -x + 1/2, y + 1/2, -z + 1/2.]

**Fig. 2.**
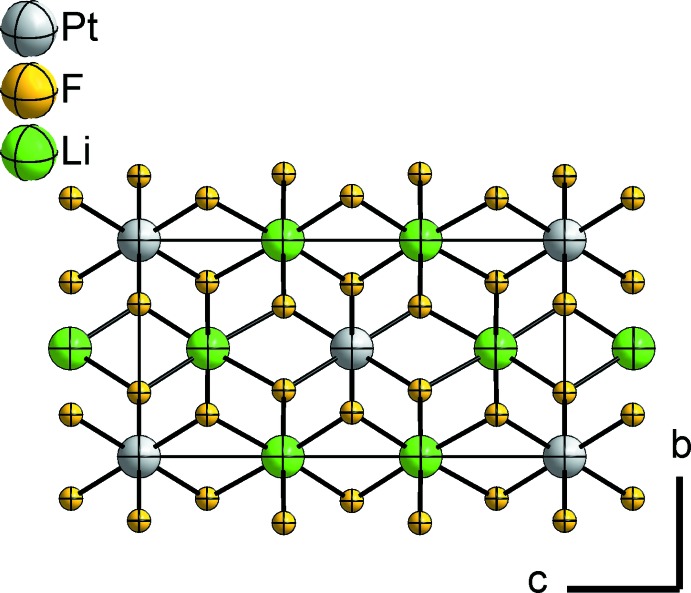
The unit cell of Li2PtF6 viewed along [100], with all atoms displayed as spheres with arbitrary radii.

## supplementary crystallographic information

### S1. Experimental

Single-crystalline Li_2_PtF_6_ was obtained by the reaction of LiF and PtF_4_ in platinum tubes. LiF was purified and dried in a stream of F_2_:Ar 1:1 at 573 K for 24 hours. PtF_4_ was synthesized according to literature procedures (Müller & Serafin, 1992). A stoichiometric mixture of the compounds was heated in a sealed platinum ampoule (jacketed in an evacuated fused silica tube) to 973 K with a rate of 30 K/d. After three weeks the ampoule was slowly cooled to room temperature and opened in an argon filled glove box. Yellow crystals of Li_2_PtF_6_ were obtained.

### S2. Refinement

The highest residual electron density is 0.85 Å from atom Pt1. Structure data have also been deposited at the Fachinformationszentrum Karlsruhe, D-76344 Eggenstein-Leopoldshafen (Germany), with depository number CSD-414496.

### Crystal data

**Table:**

Li_2_PtF_6_	*D*_x_ = 5.454 Mg m^−^^3^
*M**_r_* = 322.97	Mo *K*α radiation, λ = 0.71073 Å
Tetragonal, *P*4_2_/*m**n**m*	Cell parameters from 3453 reflections
Hall symbol: -P 4n 2n	θ = 4.4–34.6°
*a* = 4.6427 (1) Å	µ = 35.71 mm^−^^1^
*c* = 9.1234 (2) Å	*T* = 150 K
*V* = 196.65 (1) Å^3^	Cuboid, yellow
*Z* = 2	0.05 × 0.05 × 0.04 mm
*F*(000) = 276	

### Data collection

**Table:**

Oxford Diffraction Xcalibur3 diffractometer	257 independent reflections
Radiation source: Enhance (Mo) X-ray Source	184 reflections with *I* > 2σ(*I*)
Graphite monochromator	*R*_int_ = 0.062
Detector resolution: 16.0238 pixels mm^-1^	θ_max_ = 34.7°, θ_min_ = 4.5°
φ and ω scans	*h* = −7→7
Absorption correction: multi-scan (*CrysAlis RED*; Oxford Diffraction, 2007)	*k* = −7→7
*T*_min_ = 0.148, *T*_max_ = 1.000	*l* = −14→14
5829 measured reflections	

### Refinement

**Table:**

Refinement on *F*^2^	Primary atom site location: structure-invariant direct methods
Least-squares matrix: full	Secondary atom site location: difference Fourier map
*R*[*F*^2^ > 2σ(*F*^2^)] = 0.019	*w* = 1/[σ^2^(*F*_o_^2^) + (0.0246*P*)^2^ + 2.1946*P*] where *P* = (*F*_o_^2^ + 2*F*_c_^2^)/3
*wR*(*F*^2^) = 0.052	(Δ/σ)_max_ < 0.001
*S* = 1.17	Δρ_max_ = 2.51 e Å^−^^3^
257 reflections	Δρ_min_ = −1.33 e Å^−^^3^
19 parameters	Extinction correction: *SHELXL97* (Sheldrick, 2008), Fc^*^=kFc[1+0.001xFc^2^λ^3^/sin(2θ)]^-1/4^
0 restraints	Extinction coefficient: 0.041 (3)

### Special details

**Table:**

Geometry. All e.s.d.'s (except the e.s.d. in the dihedral angle between two l.s. planes) are estimated using the full covariance matrix. The cell e.s.d.'s are taken into account individually in the estimation of e.s.d.'s in distances, angles and torsion angles; correlations between e.s.d.'s in cell parameters are only used when they are defined by crystal symmetry. An approximate (isotropic) treatment of cell e.s.d.'s is used for estimating e.s.d.'s involving l.s. planes.
Refinement. Refinement of *F*^2^ against ALL reflections. The weighted *R*-factor *wR* and goodness of fit *S* are based on *F*^2^, conventional *R*-factors *R* are based on *F*, with *F* set to zero for negative *F*^2^. The threshold expression of *F*^2^ > σ(*F*^2^) is used only for calculating *R*-factors(gt) *etc*. and is not relevant to the choice of reflections for refinement. *R*-factors based on *F*^2^ are statistically about twice as large as those based on *F*, and *R*- factors based on ALL data will be even larger.

### Fractional atomic coordinates and isotropic or equivalent isotropic displacement parameters (Å^2^)

**Table:**

	*x*	*y*	*z*	*U*_iso_*/*U*_eq_	
Pt1	0.0000	0.0000	0.0000	0.0056 (2)	
F1	0.1939 (6)	0.1939 (6)	0.1599 (4)	0.0149 (7)	
F2	−0.2958 (10)	0.2958 (10)	0.0000	0.0164 (11)	
Li1	0.5000	0.5000	0.162 (2)	0.019 (4)	

### Atomic displacement parameters (Å^2^)

**Table:**

	*U*^11^	*U*^22^	*U*^33^	*U*^12^	*U*^13^	*U*^23^
Pt1	0.0054 (2)	0.0054 (2)	0.0061 (2)	0.00022 (14)	0.000	0.000
F1	0.0170 (11)	0.0170 (11)	0.0105 (13)	−0.0029 (15)	−0.0009 (10)	−0.0009 (10)
F2	0.0165 (18)	0.0165 (18)	0.016 (2)	0.004 (2)	0.000	0.000
Li1	0.024 (6)	0.024 (6)	0.010 (7)	−0.006 (7)	0.000	0.000

### Geometric parameters (Å, º)

**Table:**

Pt1—F1^i^	1.936 (4)	F1—Li1^iv^	2.062 (15)
Pt1—F1^ii^	1.936 (4)	F2—Li1^vi^	1.997 (15)
Pt1—F1^iii^	1.936 (4)	F2—Li1^vii^	1.997 (15)
Pt1—F1	1.936 (4)	Li1—F2^vi^	1.997 (15)
Pt1—F2^i^	1.942 (6)	Li1—F2^viii^	1.997 (15)
Pt1—F2	1.942 (6)	Li1—F1^ix^	2.010 (4)
Pt1—Li1^iv^	3.081 (19)	Li1—F1^x^	2.062 (15)
Pt1—Li1^v^	3.081 (19)	Li1—F1^xi^	2.062 (15)
F1—Li1	2.010 (4)	Li1—Li1^xii^	2.96 (4)
			
F1^i^—Pt1—F1^ii^	82.2 (2)	F2^vi^—Li1—F2^viii^	84.3 (8)
F1^i^—Pt1—F1^iii^	97.8 (2)	F2^vi^—Li1—F1^ix^	89.5 (4)
F1^ii^—Pt1—F1^iii^	180.0 (3)	F2^viii^—Li1—F1^ix^	89.5 (4)
F1^i^—Pt1—F1	180.0	F2^vi^—Li1—F1	89.5 (4)
F1^ii^—Pt1—F1	97.8 (2)	F2^viii^—Li1—F1	89.5 (4)
F1^iii^—Pt1—F1	82.2 (2)	F1^ix^—Li1—F1	178.8 (11)
F1^i^—Pt1—F2^i^	90.0	F2^vi^—Li1—F1^x^	176.0 (7)
F1^ii^—Pt1—F2^i^	90.0	F2^viii^—Li1—F1^x^	99.71 (17)
F1^iii^—Pt1—F2^i^	90.0	F1^ix^—Li1—F1^x^	90.5 (4)
F1—Pt1—F2^i^	90.0	F1—Li1—F1^x^	90.5 (4)
F1^i^—Pt1—F2	90.0	F2^vi^—Li1—F1^xi^	99.71 (17)
F1^ii^—Pt1—F2	90.0	F2^viii^—Li1—F1^xi^	176.0 (7)
F1^iii^—Pt1—F2	90.0	F1^ix^—Li1—F1^xi^	90.5 (4)
F1—Pt1—F2	90.0	F1—Li1—F1^xi^	90.5 (4)
F2^i^—Pt1—F2	180.00 (19)	F1^x^—Li1—F1^xi^	76.3 (7)

Symmetry codes: (i) −*x*, −*y*, −*z*; (ii) *x*, *y*, −*z*; (iii) −*x*, −*y*, *z*; (iv) *y*−1/2, −*x*+1/2, −*z*+1/2; (v) −*y*+1/2, *x*−1/2, *z*−1/2; (vi) −*x*, −*y*+1, −*z*; (vii) *x*−1, *y*, *z*; (viii) *x*+1, *y*, *z*; (ix) −*x*+1, −*y*+1, *z*; (x) *y*+1/2, −*x*+1/2, −*z*+1/2; (xi) −*y*+1/2, *x*+1/2, −*z*+1/2; (xii) −*x*+1, −*y*+1, −*z*.
